# Haeme oxygenase protects against UV light DNA damages in the retina in clock-dependent manner

**DOI:** 10.1038/s41598-017-05418-6

**Published:** 2017-07-12

**Authors:** Milena Damulewicz, Agnieszka Loboda, Alicja Jozkowicz, Jozef Dulak, Elzbieta Pyza

**Affiliations:** 1 Department of Cell Biology and Imaging, Institute of Zoology and Biomedical Research, Faculty of Biology and Earth Sciences, Jagiellonian University, 30-387 Krakow, Poland; 2 Department of Medical Biotechnology, Faculty of Biochemistry, Biophysics and Biotechnology, Jagiellonian University, 30-387 Krakow, Poland

## Abstract

In the present study, we showed that in the retina of *Drosophila*, the expression of the *ho* gene, encoding haeme oxygenase (HO), is regulated by light but only at the beginning of the day. This timing must be set by the circadian clock as light pulses applied at other time points during the day do not increase the *ho* mRNA level. Moreover, light-induced activation of HO does not depend on the canonical phototransduction pathway but instead involves cryptochrome and is enhanced by ultraviolet (UV) light. Interestingly, the level of DNA damage in the retina after UV exposure was inversely related to the circadian oscillation of the *ho* mRNA level during the night, being the highest when the HO level was low and reversed during the day. Accordingly, induction of HO by hemin was associated with low DNA damage, while inhibition of HO activity by SnPPIX aggravated the damage. Our data suggest that HO acts in the retina to decrease oxidative DNA damage in photoreceptors caused by UV-rich light in the morning.

## Introduction

Haeme oxygenase (HO) catalyses the degradation of haeme to carbon monoxide (CO), ferrous ions and biliverdin. In mammals, two HO proteins, inducible HO-1 and constitutive HO-2, are encoded by two different genes and have cytoprotective and anti-apoptotic functions by scavenging reactive oxygen species (ROS)^1^. *Drosophila* has only one gene encoding HO^2^ that plays an important role in development^3^ and in controlling the DNA damage signalling pathway^4^.

In a previous study, we found that *ho* in the fly’s retina is cyclically expressed with two peaks, 1 h after lights-on and 4 h after lights-off in a light/dark cycle^5^. This rhythm must be generated by a circadian clock because it is maintained in constant darkness (DD) and disrupted in the arrhythmic *per* ^*01*^ mutant^5^. However, HO also has an impact on the molecular mechanism of the clock^5^.

The molecular mechanism of the circadian clock is based on interlocked transcriptional-translational negative and positive feedback loops. Late in the evening, the CLOCK (CLK) protein, after reaching the appropriate level, forms dimers with CYCLE (CYC)^6^, and these heterodimers are transported into the nucleus where they bind to the promoter regulatory sequence E-box of *per*, *tim* and *ccg* (*clock*-*controlled gene*) genes, inducing their expression. At the end of the night, the amount of these gene products is sufficient to form PERIOD (PER) and TIMELESS (TIM) heterodimers^7^, and PER/TIM complexes are transported into the nucleus, where they bind to CLK/CYC and repress their activity, thereby inhibiting the transcription of their own genes *per* and *tim* ^8^. This is the main negative feedback loop of the molecular circadian clock. When *per* and *tim* transcription is inhibited, transcription of *clk* is activated, and the CLK protein is synthesized. Next, CLK and CYC form heterodimers involved in the second, positive feedback loop^6^. In the nucleus, these transcription factors bind to the E-box sequence of *vrille* (*vri*) and *par domain protein* 1 (*pdp1*) genes, activating their transcription^9, 10^. Translation and accumulation of the VRI protein in the cytoplasm occur immediately after transcription, and VRI enters the nucleus where it binds to the V/P-box sequence and represses the transcription of the *clk* gene. At the same time, *pdp1* mRNA accumulates in the cytoplasm, and translation occurs. Late at night, PDP1 is transported into the nucleus, where it activates the transcription of the *clk* gene^9, 10^. However, the *clk* mRNA cycles and CLK protein level seems to remain constant, with changes in its phosphorylation state and stability^11, 12^. Moreover, the interactions of CLK-CYC heterodimers with E-box sequences of target genes are cyclical^12^. The circadian clock is synchronized to external conditions by light and other environmental cues called Zeitgebers. The circadian clock of *D*. *melanogaster* has its own photoreceptor CRYPTOCHROME (CRY)^13^. After absorption of blue-light photons, CRY changes its conformation and binds the TIM protein. Next, CRY/TIM dimers are ubiquitinated and degraded in proteasomes. When the TIM level is reduced, PER remains in a monomeric form that is unstable and rapidly degraded^14^.

In addition to CRY, the circadian clock receives photic information from the retina of the compound eye and extraretinal photoreceptors, the H-B eyelet^15^. The retina of the fruit fly is composed of 800 ommatidia, and each of them comprises 8 types of photoreceptors (R1-R8). Six of them, R1-R6, contain Rh1 opsin, which is sensitive to a broad spectrum of light with the maximum absorption in the spectrum corresponding to green light. These photoreceptors are involved in vision, including motion perception^16^. R7 contains two different types of opsin, Rh3 and Rh4, both of which are sensitive to UV radiation. The R8 photoreceptor has Rh5 opsin, with the maximum absorption corresponding to blue light, or Rh6, which is sensitive to green light^17^. R7-R8 photoreceptors are responsible for colour and polarized light detection. The eyelet contains rhodopsin Rh5 and Rh6^18^.

The phototransduction pathway in photoreceptors is based on the visual pigment retinal. Light is absorbed by the retinal chromophore and converts rhodopsin to the active metarhodopsin state. This process catalyses the activation of a heterotrimeric G-protein (encoded by *dgq* and *gbe* genes). The GTP-GDP exchange releases the α subunit of the G protein, which activates phospholipase C (PLC), encoded by the *norpA* gene, generating inositol 1,4,5-trisphosphate (IP3) and diacylglycerol (DAG) from the phospholipid phosphatidylinositol 4,5-bisphosphate (PIP2). Light-sensitive channels TRP and TRPL, encoded by the *trp* and *trpl* genes, respectively, are activated by an unknown mechanism. Activation of TRP causes Ca^2+^ influx and membrane depolarization. PLC is a key protein in the phototransduction cascade; however, the *norpA* mutant shows a weak response to light^19, 20^. This indicates that the classical rhodopsin-dependent phototransduction pathway is not the only one active in the retinal photoreceptors of *D*. *melanogaster*.

Knowing that the *ho* mRNA level is especially high in the morning, we hypothesized that it may also be dependent on direct light exposure and that the high level of HO in the morning may protect the retina against ROS and other toxic products of phototransduction. The retina is vulnerable to light damage^21–23^, and, in mammals, it has already been suggested that HO protects the retina against light-induced degenerative processes^24, 25^. In the morning, light is rich in blue and UV light, and UV light may induce cellular degeneration^26^.

In the present study, we tested different lengths and intensities of light pulses applied at different times of the day in constant darkness (DD) and different wavelengths of light on *ho* expression. Using several clock and phototransduction mutants, we also examined the mechanisms of HO activation by light. Finally, we examined whether HO protects photoreceptors against UV-dependent degeneration.

## Results and Discussion

**Figure 1:**
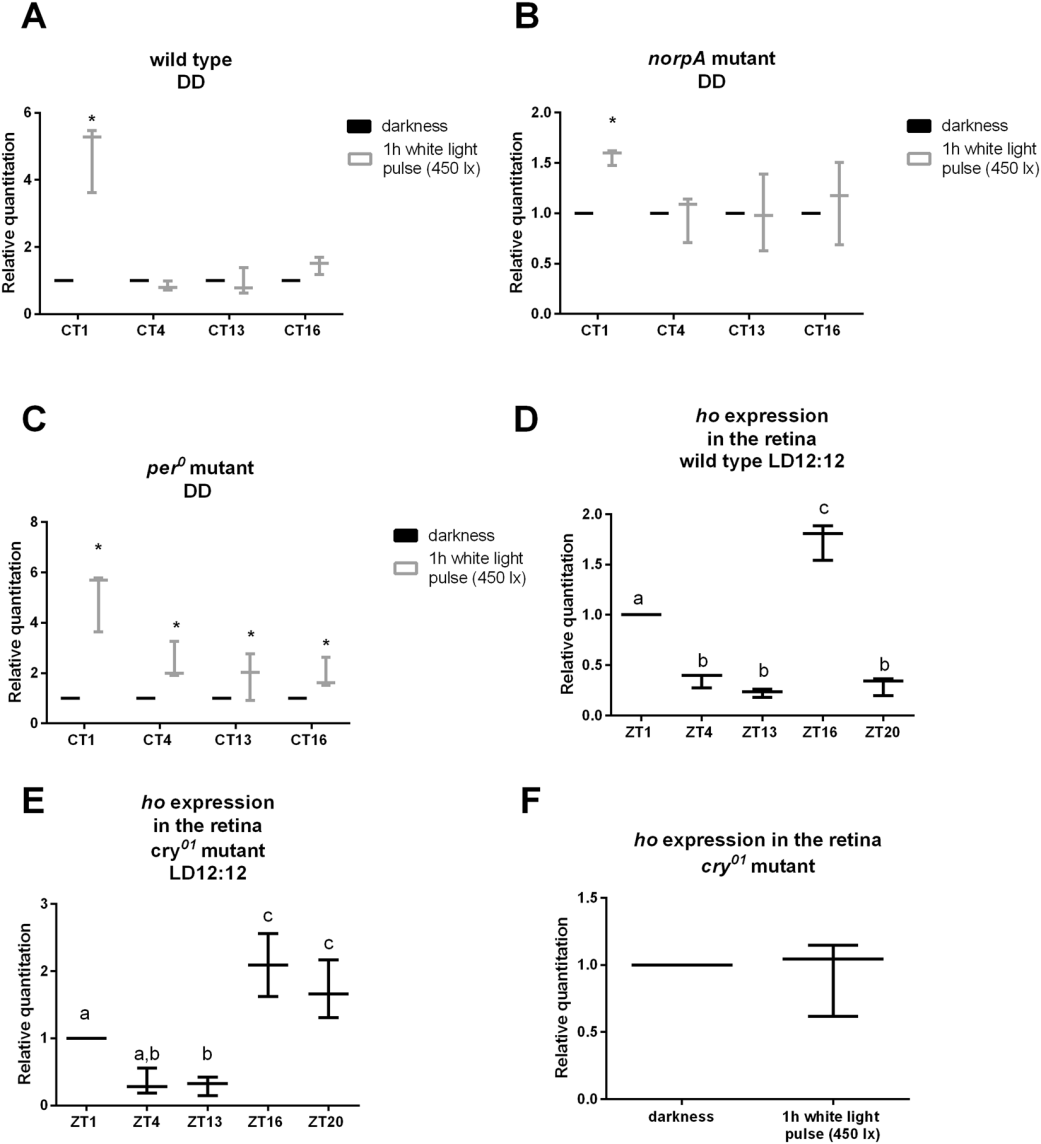
The *ho* gene mRNA level in the retina of wild type (**A**), *norpA* (**B**) and *per* ^*01*^ (**C**) mutants after 1 h light exposure in DD at CT1, CT4, CT13 or CT16. Normalization to DD (control) was done at every time point (value = 1.0, horizontal bars). Statistically significant differences are marked with asterisks. (**D**,**E**) The *ho* expression in the retina at different time points in light/dark LD12:12 in Canton S (**D**) and *cry* ^*01*^ (**F**). Data are normalized to ZT1. Statistically significant differences are marked with different letters. The same letter above the bars/plots means that there is no statistically significant differences between groups and different letters show significant differences. (**F**) The *ho* gene expression in *cry* ^*01*^ mutant 1 h after light pulse at CT0. Data are normalized to the control (DD). No statistically significant differences were found. The ends of the vertical bars for each time point represent the range of findings, with the more centrally located horizontal bars the mean.

**Figure 2:**
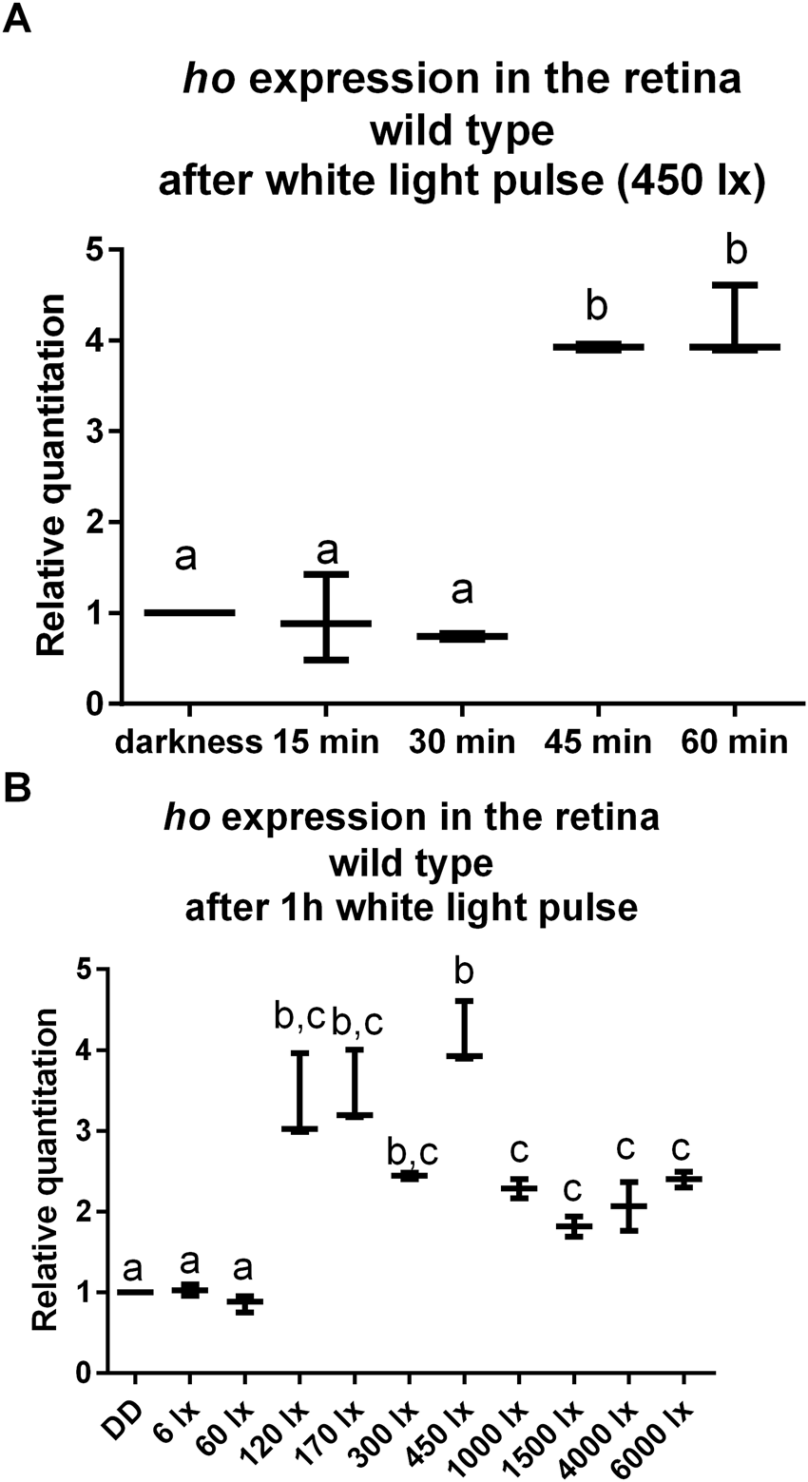
The *ho* gene mRNA level after different time (15 min, 30 min, 45 min, 60 min) of light exposure (**A**) and different light intensities (**B**) in the retina of wild type flies. Data are normalized to constant darkness (value = 1.0). Statistically significant differences are marked as different letters above bars. The ends of the vertical bars for each time point represent the range of findings, with the more centrally located horizontal bars the mean.

**Figure 3:**
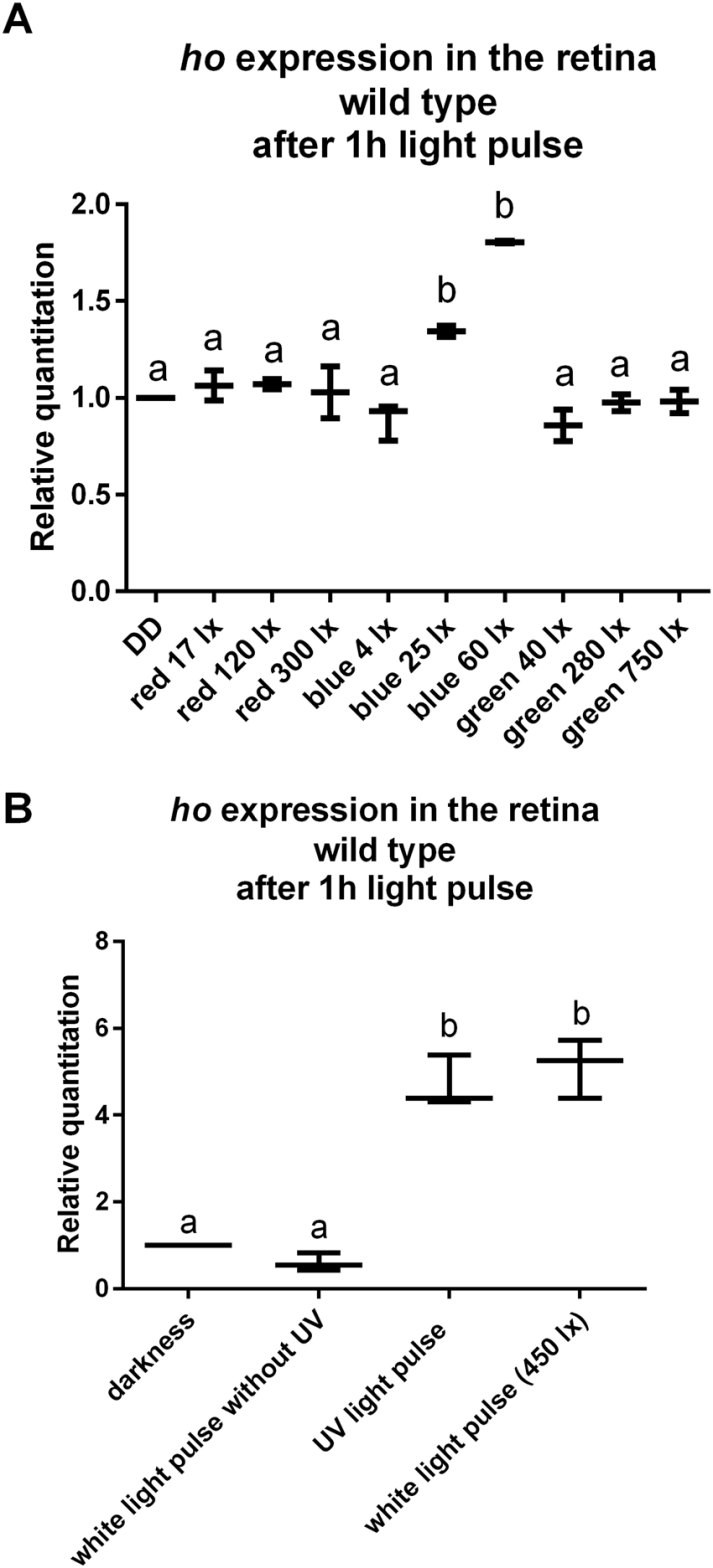
The *ho* gene mRNA level in the retina of Canton S flies after 1 h exposure to light with different wavelengths. (**A**) The following intensities were used for red light: 17, 120 and 300 lx, blue light: 4, 25 and 60 lx, green light: 40, 280 and 750 lx. (**B**) UV light and white light with filtered out UV exposures. Data are normalized to DD (value = 1.0). Statistically significant differences are marked as different letters above bars. The ends of the vertical bars for each time point represent the range of findings, with the more centrally located horizontal bars the mean.

We found that *ho* expression (measured in isolated retinas using the qPCR SybrGreen RealTime technique) was highest not only in the morning in the light/dark cycle (LD 12:12, 12 h of light and 12 h of darkness) but also after a 1-h light pulse at CT1 (1 h after the beginning of the subjective day) in constant darkness (DD) (Fig. 1). Moreover, the *ho* mRNA level increased after a white light pulse lasting for at least 45 min of 120–450 lx intensity (Fig. 2) but especially after either blue or UV light (Fig. 3). Blue light was effective at a 25–60 lx intensity, but red, green or white light without UV did not change the level of *ho* mRNA (Fig. 3). A high irradiance of white light (1000–6000 lx) caused less induction of *ho* expression than 450 lx. This observation could be explained by the saturation of photoreceptors and degradation of TIM and CRY. TIM degradation (especially in sLNvs) has been shown to depend on the light intensity, and a 10-min light pulse at 7000 nw/cm^2^ causes stronger TIM degradation than a 600-nw/cm^2^ light pulse^27^. A reduction in the TIM level may affect the transcription of clock-controlled genes, i.e., *ho* when exposed to high light intensities. On the other hand, the saturation of photoreceptors leads to an enormous increase in the calcium concentration inside photoreceptors by opening Ca^2+^-permeable TRP channels. Ca^2+^ concentration is a regulator of cell response to light because it modulates phospholipase Cβ (PLC) activity. A Ca^2+^ concentration from 10 nM to 1 µM augments PLC activity, while a higher concentration (>10 µM) suppresses it^28^. Short-term light adaptation is based on the termination of the photoresponse by Ca^2+^-dependent inhibition of TRP channels^29^. In turn, prolonged high-intensity light exposure significantly elevates the Ca^2+^ level and, in effect, the saturation. During long-term light adaptation, signalling proteins, Arrestin 2 (Arr2), Gαq and TRPL, are translocated between the cell body and rhabdomere, potentially affecting the physiology of the retina. Long-term intensive light exposure can result in a diminished photoresponse and retinal cell death because of the formation of stable, cytotoxic Rh/Arr2 complexes. In addition, the termination of the response to light is modulated by calmodulin-binding transcription factor (dCAMTA) and its target, dFbx14, which regulate processes such as rhodopsin ubiquitination^30^. Thus, it is possible that gene expression in the light-saturated retina is similar to that observed in the retina exposed to low light intensities.

The effect of light on *ho* expression at CT1 was observed in wild-type flies (Fig. 1A) and in the phototransduction mutant *norpA* that lacks PLC (Fig. 1B). In arrhythmic *per* ^*01*^ mutants, however, light activated *ho* expression at every time point studied in DD: CT1, CT4, CT13, and CT16 (CT0 and CT12 are the beginning of the subjective day and the beginning of the subjective night, respectively) (Fig. 1C). In *per* ^*01*^ mutants, *ho* expression at CT1 was still slightly higher than at other time points as in Canton S, but the differences between CT1 and the other time points were not statistically significant. This indicates that the mechanism that regulates *ho* expression is light-dependent and clock controlled but does not involve PLC, a protein of the main phototransduction pathway. Moreover, the clock blue-light photoreceptor cryptochrome (CRY)^31, 32^ is involved, as in *cry* ^*01*^ mutants, the pattern of *ho* expression oscillation was changed, and instead of two peaks, as observed in LD12:12 at ZT1 and ZT16 (Fig. 1D), the mRNA of *ho* was high at ZT16, ZT20 and ZT1. In addition, an increase in the *ho* mRNA level after light exposure was not observed in *cry* ^*01*^ mutants (Fig. 1F). This result suggests that CRY is an important element of the activation of *ho* expression in the morning. In *cry* ^*01*^ mutants, the impact of the clock on *ho* expression is maintained; however, *ho* expression is not induced by light in the morning, and its pattern is changed. The effect of light on *ho* expression is decreased because of the lack of CRY. As mentioned above, long-term light adaptation is caused by trafficking of the phototransduction proteins between the rhabdomere and the cell body. These proteins, RDGA, NINAC, and INAD, interact with CRY, and the lack of CRY in *cry* ^*01*^ mutants may change their location, function and/or modulation of the TRP channels^33^.

**Figure 4:**
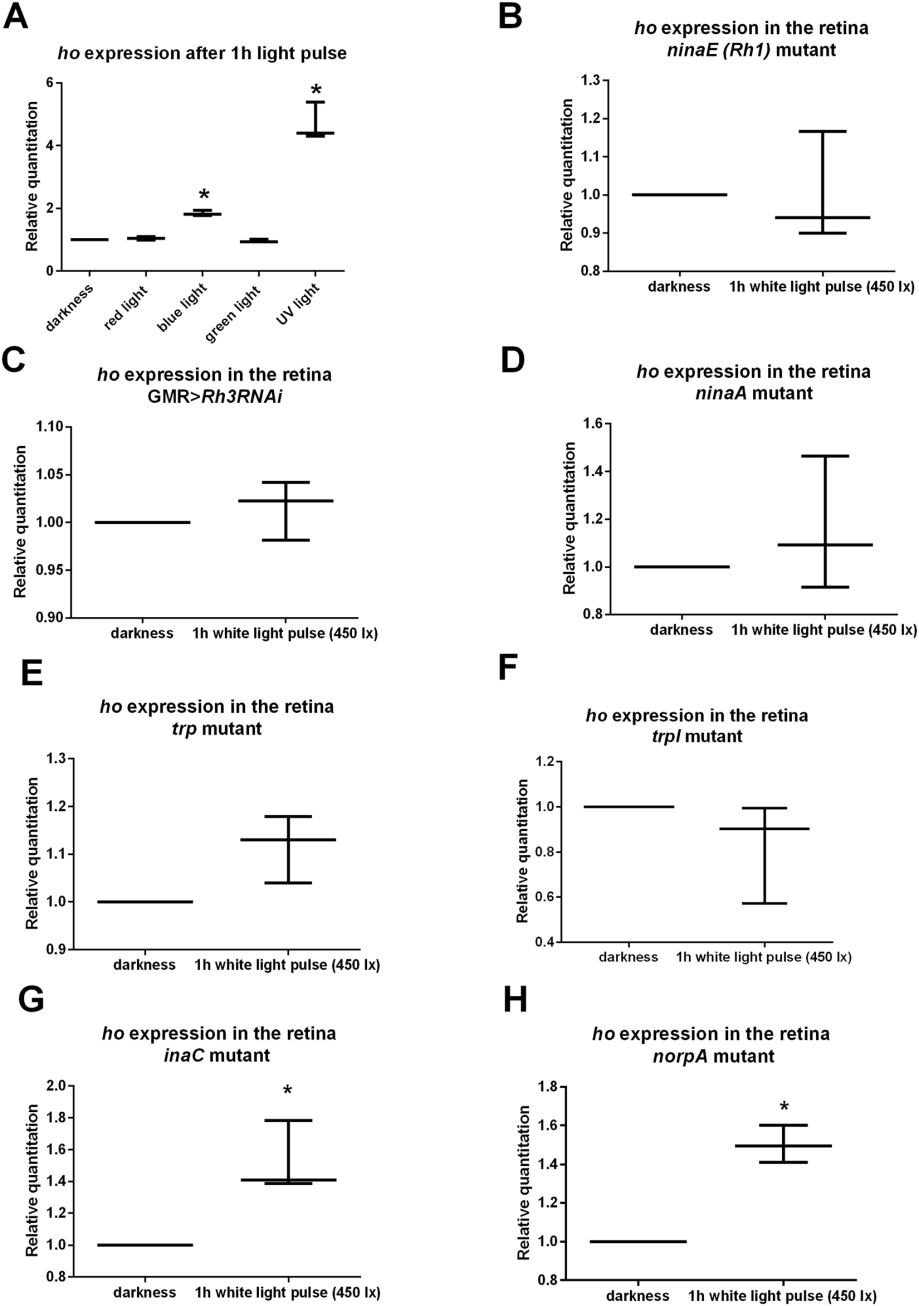
(**A**) The *ho* mRNA level in the retina after 1 h exposure to different wavelengths of light. (**B**–**H**) The *ho* mRNA level in the retina after 1 h of white light exposure. Data are normalized to DD (value = 1.0). Statistically significant differences are marked with asterisks. (**B**) GMR > *Rh3RNAi*, (**C**) *ninaE*, (**D**) *ninaA*, (**E**) *trp*, (**F**) *trpl*, (**G**) *inaC*, (**H**) *norpA*. The ends of the vertical bars for each time point represent the range of findings, with the more centrally located horizontal bars the mean.

After determining that only blue light and UV light increase *ho* mRNA level (Fig. 3), we suggested that blue light activates CRY directly in the retina and that CRY then regulates *ho* expression. A similar effect of UV light on HO-1 was also observed in mammals^34^. In addition, we found that two rhodopsins, Rh1 (sensitive to a broad spectrum of light) and Rh3 (UV-sensitive), which are expressed in R1-R6 and R7 photoreceptors, respectively^35^, contribute to this mechanism (Fig. 4B,C).

To investigate the phototransduction pathway involved in the regulation of *ho* expression in addition to CRY, we examined which proteins downstream of rhodopsins are involved in the activation of *ho* expression. Using phototransduction mutants, we found that the chaperone protein for nascent opsin NINAA, the *Drosophila* opsin Rh1 protein NINAE, and the transient receptor cationic TRP and TRPL^36^ channels are important in this process (Fig. 4C–F). However, *norpA* and *inaC*, mutants of the main phototransduction pathway proteins PLCβ and protein kinase C (PKC), respectively, do not affect *ho* expression (Fig. 4G,H). As blue Rh5- and green Rh6-sensitive rhodopsins of R8 photoreceptors, which are involved in colour vision, contribute to phototransduction in the absence of PLC, by using nonretinal PLC or alternative signalling mechanisms^37^, a similar regulation of *ho* expression by light is possible. One non-canonical phototransduction pathway is the rhodopsin/Rac2 pathway^38^; however, *ho* activation by this pathway has not been studied. Because light-induction of *ho* expression involves a non-canonical phototransduction pathway, the mechanism must converge on a common input pathway that requires Rh1, Rh3 and CRY.

**Figure 5:**
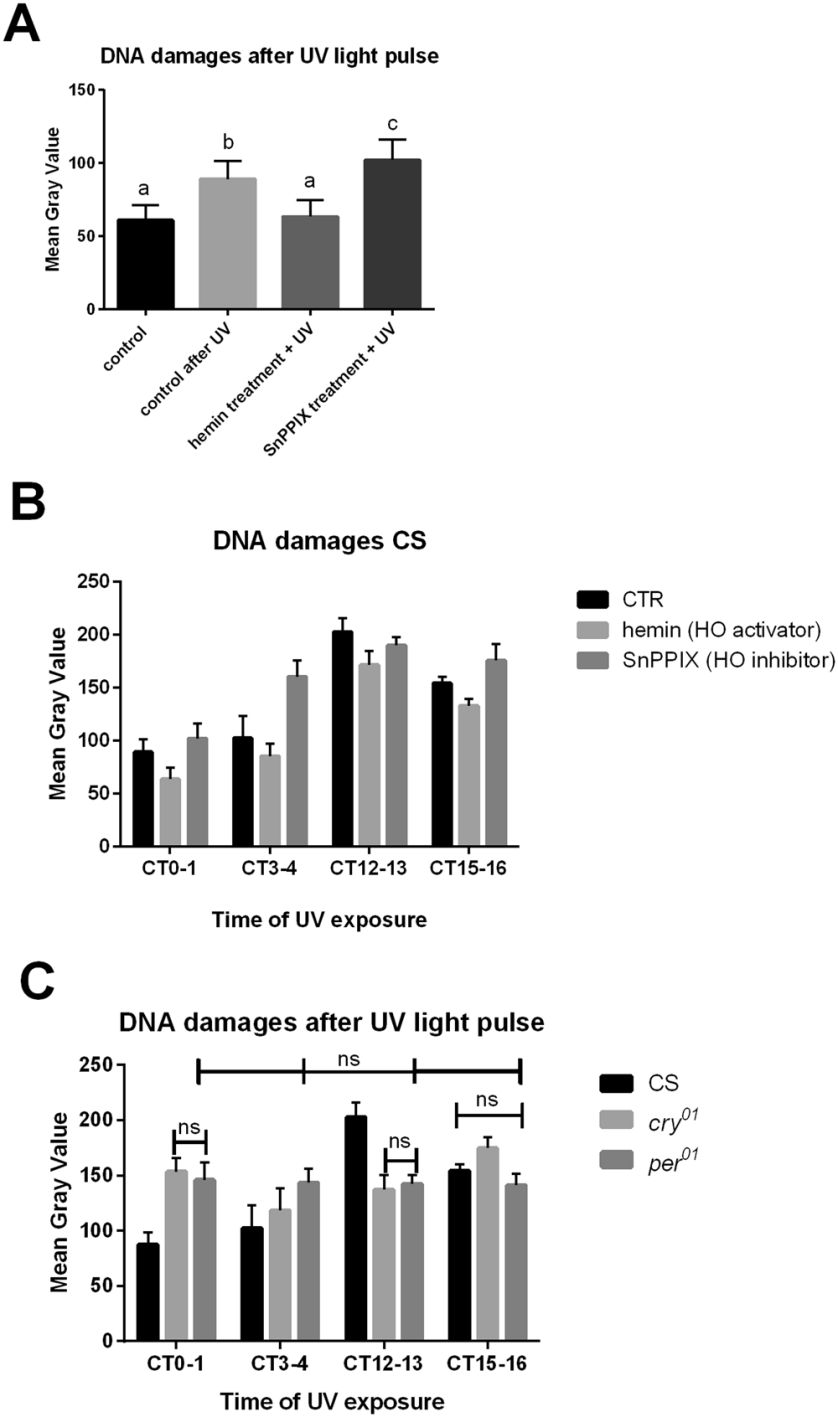
Oxidative DNA damages after UV and intense white light exposure. DNA damages were evaluated as the intensity of labeling with anti-8-hydroxyguanosine, followed by DAB/HRP enzymatic reaction and measured as Mean Gray Value using ImageJ software. (**A**) Before light exposure wild-type flies were fed a HO inhibitor (SnPPIX), HO activator (hemin) or glucose only (control 1). Flies were exposed to 1 h UV light pulse starting at CT0. One group of flies without UV treatment was used as an additional control (control 2). Statistically significant differences are shown as different letters. (**B**) Canton S (CS) flies were fed with hemin, SnPPIX or glucose (CTR) and exposed to UV light at different time points. There are statistically significant differences between CTR and experimental flies at every time point as well as between different time points in all groups of flies. (**C**) Canton S (CS), *cry* ^*01*^ and *per* ^*01*^ flies were exposed for UV light at different time points. There are statistically significant differences between CS at every time point: CT0-1 vs. CT3-4 with p < 0.01, other time points with p < 0.0001. There are statistically significant differences in *cry* ^*01*^ at every time point: CT0-1 vs. CT12-13 with p < 0.01, other time points with p < 0.0001. There are no statistically significant differences in case of *per* ^*01*^ at different time points (labeled as ns). At specific time points there are differences between: CS vs. *cry* ^*01*^ at CT0-1 (p < 0.0001), CS vs. *per* ^*01*^ at CT0-1 (p < 0.0001), CS vs. *cry* ^*01*^ at CT3-4 (p < 0.05), CS vs. *per* ^*01*^ at CT3-4 (p < 0.0001), *cry* ^*01*^ vs. *per* ^*01*^ at CT3-4 (p < 0.0001), CS vs. *cry* ^*01*^ at CT12-13 (p < 0.0001), CS vs. *per* ^*01*^(p < 0.0001) at CT12-13, CS vs. *cry* ^*01*^ at CT15-16 (p < 0.001), *cry* ^*01*^ vs. *per* ^*01*^ at CT15-16 (p < 0.0001).

Because UV and blue light can trigger reactive oxygen species (ROS)^39^, we examined DNA strand breaks in the retina photoreceptors of flies exposed to UV (1 h, 100 lx) and to high-intensity white light (3 h, 1500 lx). Our preliminary results suggested a protective role of HO in the retina against ROS^5^. Before the UV exposure, flies were fed a HO activator (hemin), inhibitor (SnPPIX), or glucose only (control 1). In hemin-treated flies, UV caused less DNA damage in the retina than in UV-exposed control flies and no more than in control (control 2) flies not treated with UV (Fig. 5A). Moreover, increased DNA damage was detected in the retina of flies treated with the HO inhibitor than in both controls and in flies treated with hemin. We also compared the DNA damage intensity at different time points and found that there were more DNA strand breaks in the retina during the night, when the HO level was low, than during the day. The most DNA breaks were observed at the beginning of the night (low level of HO) and the fewest at the beginning of the day (peak of HO) (Fig. 5B). Although *ho* mRNA levels peak in the middle of night in addition to the beginning of the day, this night peak seems to have a different function than the peak at the beginning of the day, or *ho* mRNA and HO levels increase later at night to prepare the eyes for the day. The high level of HO may also be linked to the higher damage. Degradation of haeme leads to the release of iron, which if not properly sequestered (in ferritin), can react with ROS, leading to further damage^40^.

Feeding flies with hemin and SnPPIX, which were used to activate and inhibit the activity of HO, respectively, caused similar effects at every time point studied (Fig. 5B).

*cry* ^*01*^ mutants had more DNA breaks in the retina when they were exposed to UV during the night and at the beginning of the day, and the lowest amount of DNA damage was detected during the day. There were statistically significant differences between each time point studied (Fig. 5C), suggesting that in the *cry* mutant, the rhythmic expression pattern of HO was changed, and photoreceptors were not protected against UV light at the beginning of the day. In addition, we observed more damage in *cry* ^*01*^ mutants than in wild-type flies, except at the beginning of the subjective night (CT12-13). High mortality, up to 90%, was observed in *per* ^*01*^ mutants after UV exposure. The few individuals that survived the experiment had more damage in the photoreceptors after UV exposure during the subjective day, less at CT13, and the same amount as control flies at CT16. The level of DNA breaks in *per* ^*01*^ mutants was similar at every time point studied (no statistically significant differences between time points), indicating that arrhythmic flies are not protected against damaging UV light exposure.

In conclusion, our results showed that the retina of *Drosophila* is protected against blue light and UV-rich sunlight in the morning by HO. A low level of HO in the morning, as a result of the disruption of the circadian clock, leads to an accumulation of DNA oxidative damage in the retina.

## Methods

### Animals

The following strains of *Drosophila melanogaster* were used: wild-type Canton S, *per* ^*01*^ (null mutant of the *period* gene, the main clock gene), *cry* ^*01*^ (null mutant of the *cryptochrome* gene, the circadian photoreceptor), *trp* (mutant of the *transient receptor potential* gene coding calcium channel), *trpl* (mutant of the *transient receptor potential*-*like* gene coding calcium channel), *ninaA* ^1^ (mutant of the *neither inactivation nor afterpotential A* gene coding eye-specific cyclophilin, required for Rh1 biogenesis), *ninaE* ^17^ (mutant of the *neither inactivation nor afterpotential E* gene coding rhodopsin 1), *norpA* ^7^ (mutant of the *no receptor potential A* gene coding phospholipase C), *inaC* (mutant of the *inactivation no afterpotential C* gene coding eye-specific protein kinase C), *GMR*-*Gal4* (express Gal4 factor predominantly in photoreceptors), UAS-*Rh3RNAi* (express dsRNA for the *Rh3* gene under the control of a UAS sequence). Flies were maintained under conditions of 12 h of light and 12 h of darkness (LD12:12) or in constant darkness (DD) for 5 days and at a constant temperature of 24 °C.

### Experimental procedures

#### *ho* mRNA level after light pulses during the subjective day in constant darkness (DD)

Flies were exposed to white light (450 lx) pulses of different durations: 15, 30, 45 or 60 min (Fig. 2A) and to 1-h pulses of different intensities: 6, 60, 120, 170, 300, 450, 1000, 1500, 4000, or 6000 lx (Fig. 2B). Data were normalized to the control (flies kept in darkness, value = 1). In both experiments, 30 wild-type Canton S individuals per group were used, and each experiment was repeated 3 times. For statistical analysis, we used one-way ANOVA, non-parametric Kruskal-Wallis test with multiple comparisons (comparison of the mean rank of each experimental group with that of the control group kept in darkness). Differences were considered statistically significant at p < 0.05.

#### *ho* mRNA level after light pulses applied at different times in DD

The effect of light on *ho* expression at different times of the day in DD was tested in wild-type flies and *norpA* ^7^ and *per* ^*01*^ mutants (Fig. 1A–C). One hour before CT1, CT4, CT13 or CT16 (with CT0 being the beginning of the subjective day and CT12 being the beginning of the subjective night), flies were exposed to a white light pulse (1 h, 450 lx). The control group was kept in darkness until the isolation of the retina. At each time point, 30 males were used. Data were normalized to the control kept in DD (value = 1). For statistical analysis, a two-way ANOVA nonparametric test was used, and differences were considered statistically significant at p < 0.05.

Cyclic expression of *ho* in the retina of wild-type and *cry* mutant flies in LD12:12. The *ho* mRNA level was examined in the retina of flies held in the light/dark regime LD12:12 (12 h of light and 12 h of darkness) and fixed at the following time points: ZT1 (Zeitgeber Time), ZT4, ZT13, ZT16 and ZT20 (with ZT0 being the beginning of the light/day phase and ZT12 being the beginning of the night/dark phase). Thirty wild-type Canton S and *cry* ^*01*^ flies per time point were used (Fig. 1D,E). Data were normalized to ZT1 (value = 1). The experiment was repeated 3 times. For statistical analysis, a one-way ANOVA, non-parametric Kruskal-Wallis test with multiple comparisons was used. Differences were considered statistically significant at p < 0.05.

#### *ho* mRNA level in the retina of the phototransduction mutants after light pulse in DD

The following strains, *cry* ^*01*^, *ninaE* ^17^, *ninaA* ^1^, *trp*, *trpl*, *inaC*, *norpA* ^7^ *and GMR* > *Rh3RNAi* (Figs 1F and 4B–H), were exposed to a 1-h white light pulse (450 lx), and their retina was isolated at CT1. The control group was kept in darkness. Data were normalized to control (value = 1). Thirty individuals per group were examined, and the experiment was repeated 3 times. For statistical analysis, a non-parametric Mann-Whitney test was used to compare two groups. Changes were considered statistically significant at p < 0.05.

#### *ho* mRNA level after light pulses of different wavelengths in DD

Flies were exposed to a 1-h red (17, 120, 300 lx), blue (4, 25, 60 lx), green (40, 280, 750 lx) (Fig. 3A), or UV light pulse or to a 1-h white light pulse without UV light (Fig. 3B), whereas the control group was kept in darkness. Heads were fixed at CT1. Data were normalized to the control (value = 1). Thirty individuals per group used, and each experiment was repeated 3 times. For statistical analysis, a one-way ANOVA, non-parametric Kruskal-Wallis test with multiple comparisons was used. Differences were considered statistically significant at p < 0.05.

### RNA isolation and qPCR

Males, 7 days old, were decapitated at a particular time point. Heads were fixed in 100% ethanol for 2 h, and retinas were isolated. Total RNA was isolated using TriReagent (MRC Inc.) according to the manufacturer’s protocol. The cDNA for the PCR amplification was prepared from 1 μg total RNA using Superscript II reverse transcriptase (Life Technologies) according to the manufacturer’s protocol. cDNA, diluted 1:10, was used for quantitative PCR. Each experiment was repeated at least three times. The expression of the *ho* gene was examined using SYBR Green Master Mix (Applied Biosystem) and a 7500 Fast Real-Time PCR System (Applied Biosystems). The following primers were used: *ho*, *forward primer*: 5′ACCATTTGCCCGCCGGGATG; *reverse primer*: 5′ AGTGCGACGGCCAGCTTCCT; *rpl32*, *forward primer*: 5′ AGAAGCGCAAGGAGATTGTC; *reverse primer*: 5′ ATGGTGCTGCTATCCCAATC. Product specificity was assessed by melting curve analysis, and selected samples were run on 1% agarose gels for size assessment.

Data were collected as raw C_T_ values and analysed using the 2^−ΔΔCT^ method. Gene expression was normalized on an arbitrary scale with control (as 1.0).

### Enzymatic staining

Flies were kept for 5 days in DD and starved for 6 h with water available *ad libitum*. Then, they were fed for 6 h (until decapitation) with 6% glucose in water supplemented with the HO activator, 100 μM hemin chloride (Calbiochem), or with the HO inhibitor, 100 μM tin protoporphyrin IX (SnPPIX, Frontier Scientific). The control group was fed with glucose only. All groups were next exposed to 1 h of UV light at CT0 and then to 3 h of intensive white light. After light exposure, they were decapitated and fixed in 4% paraformaldehyde. Cryosections were prepared, and immunodetection with the mouse anti-8-hydroxyguanosine primary antibody (1:500, overnight) (Acris, Cat No AM03160), which labels oxidative DNA damage, was carried out. On the next day, an HRP/DAB (ABC) detection kit was used according to the manufacturer’s protocol (Abcam, Cat No 64264). For CS control, two different groups were used: 1) those fed with glucose and exposed to UV light and intensive white light and 2) those kept in DD (Fig. 5A). The same experimental conditions (5 days in DD, feeding) were used for CS flies exposed to 1 h of UV treatment and then 3 h of white light starting at CT0, CT3, CT12 or CT15 (Fig. 5B). Clock mutants *per* ^*01*^ and *cry* ^*01*^ were exposed to UV and white light at different time points as described previously; they were not fed with the HO activator or inhibitor (Fig. 5C). DNA damage was measured as the mean grey values using ImageJ software.

### Statistical analysis

Statistical analysis was performed using two-way ANOVA, one-way ANOVA Kruskal-Wallis test or Mann-Whitney test. The GraphPad Outlier Calculator was used to eliminate outliers. GraphPad Prism 6 software was used for analysis. Differences were considered statistically significant at p < 0.05.
